# Evaluating Semi-Markov Processes and Other Epidemiological Time-to-Event Models by Computing Disease Sojourn Density as Partial Differential Equations

**DOI:** 10.1177/0272989X251333398

**Published:** 2025-05-08

**Authors:** Joachim Worthington, Eleonora Feletto, Emily He, Stephen Wade, Barbara de Graaff, Anh Le Tuan Nguyen, Jacob George, Karen Canfell, Michael Caruana

**Affiliations:** The Daffodil Centre, The University of Sydney, a joint venture with Cancer Council NSW, Sydney, NSW, Australia; The Daffodil Centre, The University of Sydney, a joint venture with Cancer Council NSW, Sydney, NSW, Australia; The Daffodil Centre, The University of Sydney, a joint venture with Cancer Council NSW, Sydney, NSW, Australia; The Daffodil Centre, The University of Sydney, a joint venture with Cancer Council NSW, Sydney, NSW, Australia; Menzies Institute for Medical Research, The University of Tasmania, Hobart, TAS, Australia; Menzies Institute for Medical Research, The University of Tasmania, Hobart, TAS, Australia; WHO Collaborating Centre for Viral Hepatitis, The Peter Doherty Institute for Infection and Immunity; Storr Liver Centre, The Westmead Institute for Medical Research, Westmead Hospital and University of Sydney, Sydney, NSW, Australia; School of Public Health, Faculty of Medicine and Health, The University of Sydney, Sydney, NSW, Australia; The Daffodil Centre, The University of Sydney, a joint venture with Cancer Council NSW, Sydney, NSW, Australia

**Keywords:** cost-effectiveness analysis, Markov models, probabilistic sensitivity analysis, simulation methods, survival analysis, semi-markov models, time-to-event modelling

## Abstract

**Introduction:**

Epidemiological models benefit from incorporating detailed time-to-event data to understand how disease risk evolves. For example, decompensation risk in liver cirrhosis depends on sojourn time spent with cirrhosis. Semi-Markov and related models capture these details by modeling time-to-event distributions based on published survival data. However, implementations of semi-Markov processes rely on Monte Carlo sampling methods, which increase computational requirements and introduce stochastic variability. Explicitly calculating the evolving transition likelihood can avoid these issues and provide fast, reliable estimates.

**Methods:**

We present the sojourn time density framework for computing semi-Markov and related models by calculating the evolving sojourn time probability density as a system of partial differential equations. The framework is parametrized by commonly used hazard and models the distribution of current disease state and sojourn time. We describe the mathematical background, a numerical method for computation, and an example model of liver disease.

**Results:**

Models developed with the sojourn time density framework can directly incorporate time-to-event data and serial events in a deterministic system. This increases the level of potential model detail over Markov-type models, improves parameter identifiability, and reduces computational burden and stochastic uncertainty compared with Monte Carlo methods. The example model of liver disease was able to accurately reproduce targets without extensive calibration or fitting and required minimal computational burden.

**Conclusions:**

Explicitly modeling sojourn time distribution allows us to represent semi-Markov systems using detailed survival data from epidemiological studies without requiring sampling, avoiding the need for calibration, reducing computational time, and allowing for more robust probabilistic sensitivity analyses.

**Highlights:**

- Time-inhomogeneous semi-Markov models and other time-to-event–based modeling approaches can capture risks that evolve over time spent with a disease.
- We describe an approach to computing these models that represents them as partial differential equations representing the evolution of the sojourn time probability density.
- This sojourn time density framework incorporates complex data sources on competing risks and serial events while minimizing computational complexity.

## Graphical abstract

**Graphical Abstract:**
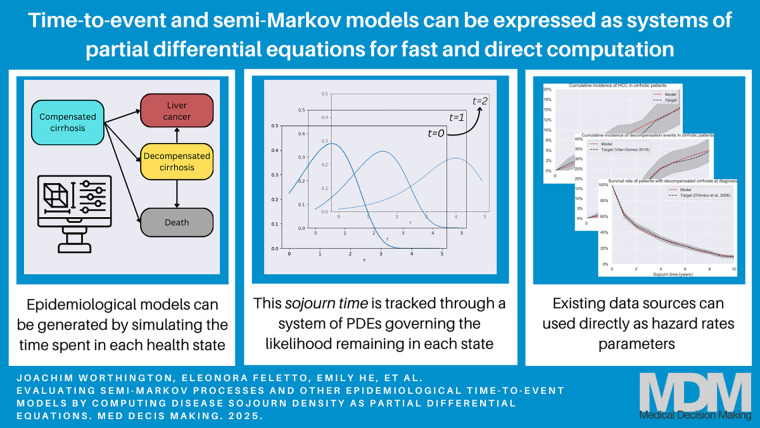
This is a visual representation of the abstract.

Health economic modeling evaluates the impact and cost-effectiveness of interventions by synthesizing and extrapolating data on health and cost outcomes. Almost all health economic models are state-transition models,^1^ capturing a set of discrete health states an individual can be in and the likelihood of transitioning between these states. These models can simulate health care scenarios to predict outcomes, aiding informed decision making in resource allocation. There are various mathematical and computational approaches to state-transition modeling, each with their own strengths and limitations.^2^

Many health economic analyses use Markov models, which are easy to comprehend, implement, and compute, and they often have high parameter identifiability (i.e., transition rates can be supported by the available data) due to the clear delineation of health states. However, Markov models have notable disadvantages. The Markov property restricts the likelihood of a state transition to be either constant (time homogeneous) or dependent on only the current model time (time inhomogenous); they cannot depend on the time spent in the current state (the sojourn time). Consequently, shorter sojourn times for any particular state transition at a given time are more likely than any longer times.^3^ While this may be addressed with the addition of hidden states, this can lead to serious problems such as overfitting^4^ due to lack of identifiability,^5^ leading to poorly fitted models and spurious findings. A poor choice of time discretization/cycle length^6^ can also dramatically bias a Markov model’s outcomes, and altering the cycle length can lead to numerical errors.^7^

Semi-Markov models (or equivalently, Markov renewal processes)^8–10^ can capture significantly more detail than Markov models can, allowing the time between state transitions to vary according to any distribution^11^ rather than being constrained to a geometric distribution. This can dramatically increase model fidelity. This means the risk of developing a disease can depend directly on the length of time spent with a precursor condition,^12,13^ the sojourn time. Detailed survival analyses from prior studies can be used directly to inform disease risk, which has been identified as a priority in health technology assessments.^14,15^ This approach conserves identifiability by explicitly including states for which survival data are available. By further generalizing to time-inhomogeneous semi-Markov processes,^11^ transitions can change over time, allowing the model to also capture risks that vary by age and secular trends. Semi-Markov models are also used in fields such as actuarial science^16–18^ and engineering.^19^ For example, semi-Markov models of cancer control can be used to increase the fidelity of estimates of disease progression and the impact of interventions.^20,21^

Unlike Markov models, the dynamics of semi-Markov models cannot usually be analytically computed. Instead, Monte Carlo sampling methods are typically used to estimate patient-level dynamics.^22–24^ In this formulation, the semi-Markov model will describe a patient-level simulation approach^25^ equivalent to a microsimulation or discrete event simulation,^26–29^ which allows for a significant level of depth and detail.^2,30^ However, Monte Carlo sampling introduces stochastic uncertainty to the model (first-order uncertainty).^25,31^ In general, there is no consistent method of estimating the number of samples required to estimate a particular outcome. As a first-order approximation, if outcomes are assumed to be binomially distributed by the model, the length of confidence interval for the outcome is approximately $O\left(1/\sqrt{N}\right)$ , meaning an increasing number of simulations is required to accurately estimate an outcome, particularly for rare events. This can dramatically increase the computation time required for modeling, which both slows model development and limits the ability to conduct extensive sensitivity analyses, a priority for health economic estimates.^32^ The tradeoff between discrete-time Markov-type models and continuous-time semi-Markov simulation models can lead to many potential sources of bias and inaccuracy.^33^

We developed the sojourn density model framework for explicit evaluation of semi-Markov and related models. This approach models the dynamics of the probability density of the sojourn time as a system of partial differential equations (PDEs), tracking both the likelihood of being in a given state and the time spent in that state, avoiding the need for stochastic sampling methods. The modeling framework is parametrized by the cause-specific hazard rates^34,35^ (analogous to transition intensities^36^ or the force of increment^37^), allowing us to exploit survival data and methods^38^ such as Kaplan–Meier estimators^34,39,40^ and risk ratios.^41^ This captures the same detail as survival models, with the addition of serial state transitions. This approach is adapted from existing methods used in actuarial science,^11,16–18^ which use the Kolmogorov forward equation.^42^ We have modified this for an epidemiology and health economic context, including adapting the notation and simplifying the construction where possible.

In this study, we describe the model structure for sojourn density models, compare their strengths and weaknesses versus other modeling approaches, and describe a numerical scheme for calculating these models. To demonstrate the advantages of this approach, we include an illustrative model of liver disease. In Australia, liver cancer is the seventh most common cause of cancer-related death,^43^ with increasing incidence and mortality trends.^44^ Routine surveillance of patients with liver cirrhosis (late-stage liver scarring) can increase detection at early stages and improve survival^45^ but requires an understanding of the competing risk of liver cancer versus other-cause mortality. Our modeling approach allows us to incorporate competing risks of decompensation, cancer, and other-cause mortality and complex survival data,^46,47^ which have been noted as key considerations in liver disease modeling.^48^

## Methods

We now describe the sojourn density model framework and demonstrate its useful mathematical properties. We also develop a numerical scheme for the calculation of these models.

### Sojourn Density Model

Consider a state transition model with N possible discrete states labeled 1,2,...,N , such that at any time t∈T⊂R (typically T=[0,∞) ), an individual is in exactly 1 of these states. We not only describe the likelihood of an individual being in a given state but also track the distribution of the length of time spent in that state, the sojourn time τ∈T .

Doing so defines a continuous-time random process $\{X_{t}{\}}_{t\in T}$ with discrete state space {1,2,...,N} such that $P\left(X_{t}=i\right)=P\left(\mathrm{individual}\;\mathrm{in}\;\mathrm{state}\;i\;\mathrm{at}\;\mathrm{time}\;t\right)$ . We can equivalently define the jump process $\{Y_{n},T_{n}{\}}_{n=0,1,2,\ldots }$ such that $Y_{n}=X_{t}\;\forall \;t\in [T_{n},T_{n+1})$ , $Y_{n}\neq Y_{n+1}$ .^49^

The cause-specific hazard rates

(1) $$\lambda_{i,j}\left(t,\tau \right),$$

depending on both the model time t and sojourn time τ , are the key parameters for the model. These are the instantaneous transition rates for an individual in state i to transition to state j at time t , given that the individual has so far spent time τ in i without any transitions (i.e, entered state i at time t−τ ). Hazard rates are widely used parameters in public health modeling.^22^ In some cases, h is used for hazard rates.^50^ They are equivalent to the force of increment discussed in equation 4.6 of Hoem^11^ in the case s=t (i.e., the reference time when the model is in state i is the same as the start of the period over which the transition may occur).

As the transition likelihoods depend on only the current state and the sojourn time, the random process $X_{t}$ is a semi-Markov proccess,^36^ as the sequence of transitions is Markov, but the time between transitions is not neccesarily exponentially distributed. By allowing the hazard rate to depend on t , this generalizes to time-inhomogeneous semi-Markov models. This t dependency can capture temporal trends as well as evolving risk with age $a=a_{0}+t$ , where $a_{0}$ is the age at t=0 . While $X_{t}$ is a semi-Markov process, the jump process $\left(Y_{n},T_{n}\right)$ is a Markov renewal process.^51^

For the hazard rate $\lambda_{i,j}\left(t,\tau \right)$ to represent the transition rate from state i to state j at time t given sojourn time τ , by equation (3) in Król et al.,^22^ the following should hold:

(2) $$\begin{array}{c} \lambda_{i,j}\left(t,\tau \right)=\underset{\Delta t\to 0}{\lim}\frac{1}{\Delta t}P(Y_{n+1}=j,\;\\ \;\;\;\;\;\;T_{n+1}\in [t,t+\Delta t)\;|Y_{n}=i,T_{n}=t-\tau )\\ \;\;\;=\underset{\Delta t\to 0}{\lim}\frac{1}{\Delta t}P\left(X_{t+\Delta t}=j\;|\;X_{s}=i\;\forall \;s\in [t-\tau ,t\right),\;X_{t-\tau }\neq i). \end{array}$$

For convenience, assume $\lambda_{i,i}\left(t,\tau \right)=0$ (i.e., there are no self-loops). If there is no possible transition between i and j , then $\lambda_{i,j}=0$ .

To track the model state and the sojourn time, we introduce the dynamic density functions for the sojourn time:

(3) $$f_{i}\left(t,\tau \right):T\times T\to [0,\infty )$$

for i=1,2,…,N . For a fixed time t∈T , these are probability density functions on the space of sojourn times and states (τ,i)∈T×{1,2,...N} , describing the likelihood of being in a particular state with a particular sojourn at the present time. This notation is based on the notation introduced by Asanjarani et al.,^36^ who described an equivalent density function with a single time/sojourn time variable.

Then we can define the process $X_{t}$ by the total probability mass in that state:

(4) $$\begin{array}{c} \begin{array}{c} P\left(X_{t}=i\right)=\int_{0}^{\infty }f_{i}\left(t,s\right)\mathrm{ds}=:g_{i}\left(t\right). \end{array} \end{array}$$

We should expect the total probability mass to be 1, that is,

(5) $$\sum_{i}g_{i}\left(t\right)=1.$$

Our goal now is to describe the dynamics of $f_{i}\left(t,\tau \right)$ and then show this definition satisfies (2) and (5). Let $f_{i}\left(t,\tau \right)$ satisfy the partial differential equation

(6) $$\frac{\partial }{\partial t}f_{i}\left(t,\tau \right)+\frac{\partial }{\partial \tau }f_{i}\left(t,\tau \right)=-\left(\sum_{j}\lambda_{i,j}\left(t,\tau \right)\right)f_{i}\left(t,\tau \right)$$

with boundary condition

(7) $$f_{i}\left(t,0\right)=\sum_{j}\left(\int_{0}^{\infty }\lambda_{j,i}\left(t,\tau \right)f_{j}\left(t,\tau \right)d\tau \right).$$

This equation is equivalent to the Kolmogorov forward equation.^17,42^ The left-hand side of (6) represents the transport equation along lines of constant t−τ , representing the likelihood of remaining in state i while both t and τ increase, whereas the right-hand side captures the likelihood of making a transition to a subsequent state. Along the directional derivative $\nabla_{\left(1,1\right)}f_{i}$ , the likelihood decays proportionally to the hazard rate (see the “Method of Characteristics and Survival Analysis” section). More rigorous derivations of this PDE are included elsewhere.^16,17^ The findings here have been simplified for a health economic context and assume the hazard rates $\lambda_{i,j}\left(t,\tau \right)$ are smooth.

The boundary condition (7) represents the accumulation of probability mass in state i post-transition. By (5), the initial conditions $f_{i}\left(0,\tau \right)$ should satisfy

(8) $$\sum_{i}\int_{0}^{\infty }f_{i}\left(0,\tau \right)d\tau =1.$$

In addition,

(9) $$\underset{\tau \to \infty }{\lim}f_{i}\left(t,\tau \right)=0$$

(i.e., supported on finite time).

Compare (6) to equation (2.37.1) in Helwich.^17^ Helwich considered the likelihood $p_{ij}\left(s,t,u,v\right)$ of transition from state y to state z such that $X_{s}=i$ with sojourn time u and $X_{t}=j$ with sojourn time less than v . In our notation, this is equivalent to fixing the model at state i with sojourn time u at time s (i.e., $f_{k}\left(s,\tau \right)=\delta \left(\tau -u\right)$ if k=i else 0 ), then tracking the evolution of $f_{j}\left(t,\tau \right)$ for t in a small neighbourhood of s .

We now have a complete description of the random process $X_{t}$ through (4), (6), (7) and appropriate initial conditions. This constitutes what we will call a *sojourn density model*. With this current definition, sojourn density models are a type of semi-Markov model as a specific parametrization using hazard rates and computed through the sojourn density functions. In the sections “Sojourn-Preserving Transitions” and “Inhomogeneous Hazard Rates and Infectious Disease Modeling,” we introduce modifications that expand the sojourn density framework to include some non-semi-Markov models.

We now show that the process defined by (4) is well-defined and driven by the hazards $\lambda_{i,j}\left(t,\tau \right)$ as expected.

**Theorem 2.1.** The random process defined by (4) with dynamics defined by (6) and (7) satisfies the conditions (2) and (5).

*Proof.* The boundary condition (7) can be justified by calculating the derivative of $g_{i}\left(t\right)$ , that is, the rate of change in likelihood of being in state i at time t :

(10) $$\begin{array}{c} \frac{d}{\mathrm{dt}}g_{i}\left(t\right)=\frac{d}{\mathrm{dt}}\int_{0}^{\infty }f_{i}\left(t,\tau \right)d\tau \\ =\int_{0}^{\infty }\frac{\partial }{\partial t}f_{i}\left(t,\tau \right)d\tau \\ =-\int_{0}^{\infty }\frac{\partial }{\partial \tau }f_{i}\left(t,\tau \right)d\tau -\int_{0}^{\infty }\left(\sum_{j}\lambda_{i,j}\left(t,\tau \right)\right)f_{i}\left(t,\tau \right)d\tau \\ =f_{i}\left(t,0\right)-\int_{0}^{\infty }\left(\sum_{j}\lambda_{i,j}\left(t,\tau \right)\right)f_{i}\left(t,\tau \right)d\tau \\ =\sum_{j}\left(\underset{\mathrm{entering}\;\mathrm{state}\;i}{\underset{︸}{\int_{0}^{\infty }\lambda_{j,i}\left(t,\tau \right)f_{j}\left(t,\tau \right)d\tau }}\right)-\int_{0}^{\infty }\left(\underset{\mathrm{leaving}\;\mathrm{state}\;i}{\underset{︸}{\sum_{j}\lambda_{i,j}\left(t,\tau \right)}}\right)f_{i}\left(t,\tau \right)d\tau . \end{array}$$

This satisfies the definition of the hazard rate: the likelihood of being in state i decreases proportionally to the hazard of every transition leaving state $i\;\sum_{j}\lambda_{i,j}\left(t,\tau \right)$ and increases for every transition entering state $i\;\sum_{j}\lambda_{j,i}\left(t,\tau \right)$ proportional to the weight of those states $f_{j}\left(t,\tau \right)$ . In addition,

(11) $$\frac{d}{\mathrm{dt}}\left(\sum_{i}g_{i}\left(t\right)\right)=0,$$

that is, that the total probability mass is conserved. Therefore, with initial conditions satisfying equation (8) and boundary conditions (7), (5) is satisfied.

The propagation of hazards (2) through (6) can be observed through analogy with corollary 2.37 in Helwich^17^ as noted above; a proof in the current notation is included in Appendix A.    ■

### Method of Characteristics and Survival Analysis

**Figure 1:**
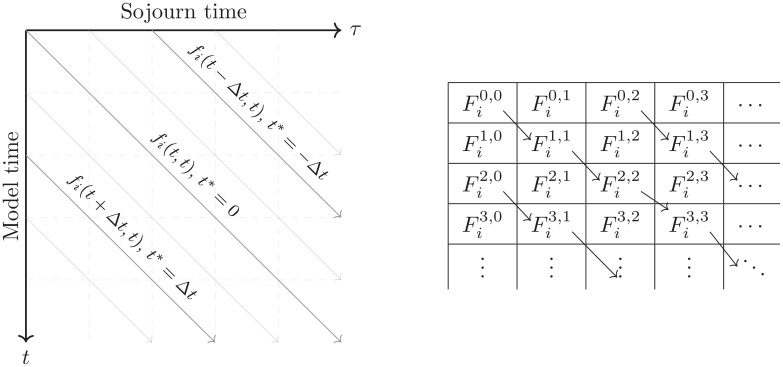
Characteristics of constant $t^{*}=t-\tau$ for $f_{i}\left(t,\tau \right)$ (left) and the corresponding elements $F_{i}^{m,n}$ with constant m−n for the numerical scheme (right).

We now compute the survival curves using method of characteristics. We refer to *survival* in the sense of the time until an event of interest, which may or may not be mortality, as is often used in epidemiological analyses.^34,52^ The use of characteristics is equivalent to the approach in section 2.5 of Buchardt et al.^16^ or the explicit solution in equation 9 from Asanjarani et al.^36^ As noted, equation (6) is best interpreted along characteristics of constant t−τ corresponding to the state entry time (Figure 1). This allows explicit solutions through method of characteristics as well as analogies to other survival modeling approaches.

Consider the characteristic $t=t^{*}+\tau$ for fixed $t^{*}\in R$ (Figure 1). Then, along this characteristic,

(12) $$\frac{d}{d\tau }f_{i}\left(t^{*}+\tau ,\tau \right)\;=\frac{\partial }{\partial t}f_{i}\left(t^{*}+\tau ,\tau \right)\frac{d}{d\tau }\left(t^{*}+\tau \right)+\frac{\partial }{\partial \tau }f_{i}\left(t^{*}+\tau ,\tau \right)$$

(13) $$=-\left(\sum_{j}\lambda_{i,j}\left(t^{*}+\tau ,\tau \right)\right)f_{i}\left(t^{*}+\tau ,\tau \right).$$

If $t^{*}>0$ , then t>τ , which corresponds to a characteristic starting from the boundary τ=0 ; if $t^{*}\leq 0$ , then t≤τ , which corresponds to a characteristic from the initial condition t=0 .

Defining $t_{0}=\max\left(t^{*},0\right)$ , $\tau_{0}=\max\left(-t^{*},0\right)$ (so that $t^{*}=t_{0}-\tau_{0}$ , $t_{0},\tau_{0}\geq 0$ ), the initial condition (at the observable start of the characteristic; not necessarily at t=0 ) is at $\left(t_{0},\tau_{0}\right)$ and so (12) has solution

(14) $$f_{i}\left(t^{*}+\tau ,\tau \right)=f_{i}\left(t_{0},\tau_{0}\right)e^{-\lambda_{i}\left(t^{*}+\tau ,\tau \right)}$$

where

(15) $$\lambda_{i}\left(t,\tau \right)=\sum_{j}\left(\int_{\tau_{0}}^{\tau }\lambda_{i,j}\left(t-\tau +s,s\right)\mathrm{ds}\right).$$

The function $\lambda_{i}\left(t,\tau \right)$ is the *overall cumulative hazard*^41^ along the characteristic $t-\tau =t^{*}$ . Then,

(16) $$S_{i}\left(t,\tau \right)=e^{-\lambda_{i}\left(t,\tau \right)},$$

is the *overall survival* (i.e., the likelihood no transitions occur) along this characteristic, and $f_{i}\left(t^{*}+\tau ,\tau \right)=f_{i}\left(t_{0},\tau_{0}\right)S_{i}\left(t^{*}+\tau ,\tau \right)$ .

We can correspondingly calculate the time-to-event distribution after entering state i at time t−τ as the distribution

(17) $$\lambda_{i,j}\left(t,\tau \right)S_{i}\left(t,\tau \right)$$

across j=1,2,…,N . This allows translation to time-to-event epidemiological models and access to time-to-event methodology.^35,53,54^

It follows that the probability of the next transition being from i→j before time $t=t^{*}+\tau$ given entry to state i at time $t_{0}$ , is

(18) $$\begin{array}{c} \begin{array}{c} P(\mathrm{next}\;\mathrm{transition}\;\mathrm{time}\;<t\;\;\cap \;\mathrm{next}\;\mathrm{transition}\;i\to j\;|\;\\ \mathrm{entered}\;\mathrm{state}\;i\;\mathrm{at}\;\mathrm{time}\;t_{0})\\ \;=P\left(Y_{n+1}=j,\;T_{n+1}<t\;|\;Y_{n}=i,\;T_{n}=t_{0}\right)\\ \;=\int_{t_{0}}^{t}\lambda_{i,j}\left(s,s-t^{*}\right)S_{i}\left(s,s-t^{*}\right)\mathrm{ds}. \end{array} \end{array}$$

The limit of this is the likelihood of the next transition being to a particular state:

(19) $$\begin{array}{c} P(\mathrm{next}\;\mathrm{transition}\;i\to j\;|\;\mathrm{entered}\;\mathrm{state}\;i\;\mathrm{at}\;\mathrm{time}\;t_{0})\\ \;=\int_{t_{0}}^{\infty }\lambda_{i,j}\left(s,s-t^{*}\right)S_{i}\left(s,s-t^{*}\right)\mathrm{ds}. \end{array}$$

We can also calculate the cause-specific survival, assuming other transitions (or events) are censored:

(20) $$S_{i,j}\left(t,\tau \right)=e^{-\int_{\tau_{0}}^{\tau }\lambda_{i,j}\left(t-\tau +s,s\right)\mathrm{ds}}.$$

Where cause-specific survival data (equivalently, cause-specific cumulative incidence) is available with competing events censored, this may provide a calibration target for the hazard rates, which can be directly identified from the survival model (see “Hazard and Cumulative “Hazard in Clark et al.^34^); parametrization of $\lambda_{i,j}\left(t,\tau \right)$ across $t^{*}$ can then be identified.

Compare this with the common approach in analytical epidemiological models in which the likelihood of transition is computed explicitly as the convolution of the τ -dependent hazard rate and the time of entry.^55–57^ In our formulation, this is reflected in the boundary conditions

(21) $$\begin{array}{c} \begin{array}{c} f_{i}\left(t,0\right)=\sum_{j}\left(\int_{0}^{\infty }\lambda_{j,i}\left(t,\tau \right)f_{j}\left(t,\tau \right)d\tau \right)\\ \;=\sum_{j}\left(\int_{0}^{\infty }f_{j}\left(t-\tau ,0\right)\lambda_{j,i}\left(t,\tau \right)S_{j}\left(t,\tau \right)d\tau \right). \end{array} \end{array}$$

If we define entry time distribution $F_{j}\left(x\right)=f_{j}\left(x,0\right)$ and survival-adjusted cause-specific hazard rate $H_{\left(j,i\right)}\left(x\right)=\lambda_{j,i}\left(t,x\right)S_{j}\left(t,x\right)$ , then $F_{i}\left(t\right)=\sum_{j}\left(F_{j}*H_{j,i}\right)\left(t\right)$ , the convolution of these. Our approach generalizes this to incorporate serial transitions.

### Initial Conditions

The appropriate initial conditions are highly dependent on the system being modeled. The Dirac delta function around τ=0 is a potential initial condition, corresponding to starting in state i with no existing sojourn time; explicitly,

(22) $$f_{j}\left(0,\tau \right)=\left\{ \begin{array}{cc} \delta \left(\tau \right) & \mathrm{if}\;i=j\\ 0 & \mathrm{if}\;i\neq j. \end{array}\right.$$

In practice, modeling may start in situ, that is, after some sojourn time, informing a distribution for the initial condition. For example, this could reflect the time an individual would have spent in a health state by the start of a trial (“Incorporating Lead-Time Biases” section).

### Numerical Scheme

The characteristic solutions developed in the “Method of Characteristics and Survival Analysis” section can be used to develop a numerical scheme. For a discretization of T by fixed time steps $\Delta t\in R^{+}$ , define

(23) $$\begin{array}{c} \begin{array}{c} F_{i}^{m,n}:=f_{i}\left(m\Delta t,n\Delta t\right)\\ \;=\left\{ \begin{array}{c} f_{i}\left(\left(m-n\right)\Delta t,0\right)e^{-\lambda_{i}\left(m\Delta t,n\Delta t\right)}\;\;\mathrm{for}\;m\geq n\\ f_{i}\left(0,\left(n-m\right)\Delta t\right)e^{-\lambda_{i}\left(m\Delta t,n\Delta t\right)}\;\;\mathrm{for}\;m<n \end{array}\right.\\ \;=\left\{ \begin{array}{c} F_{i}^{m-n,0}e^{-\lambda_{i}\left(m\Delta t,n\Delta t\right)}\;\;\mathrm{for}\;m\geq n\\ F_{i}^{0,n-m}e^{-\lambda_{i}\left(m\Delta t,n\Delta t\right)}\;\;\mathrm{for}\;m<n \end{array}\right. \end{array} \end{array}$$

for $m,n\in {\mathbb{Z}}^{+}$ . Each value of m−n∈Z corresponds to a characteristic with $t^{*}=\left(m-n\right)\Delta t$ . For m<n , the initial condition is $F_{i}^{0,n-m}=f_{i}\left(0,\left(n-m\right)\Delta t\right)$ . For m≥n , the initial condition $F_{i}^{m-n,0}=f_{i}\left(\left(m-n\right)\Delta t,0\right)$ is the boundary condition of the full PDE.

The cumulative hazard functions $\lambda_{i}\left(t,\tau \right)$ may be computed by (15) in simple cases or otherwise approximated. Equivalently, each $F_{i}^{m,n}$ element can be calculated iteratively by

(24) $$F_{i}^{m,n}=F^{m-1,n-1}e^{-\sum_{j}\int_{0}^{\Delta t}\lambda_{i,j}\left(\left(m-1\right)\Delta t+s,\left(n-1\right)\Delta t+s\right)\mathrm{ds}}.$$

(see Figure 1 for an illustration of this; compare Buchardt et al.^16^). In many cases, the integral in (24) can be explicitly computed; otherwise, it can be approximated by the midpoint $\int_{0}^{\delta t}\lambda_{i,j}\left(\left(m-1\right)\Delta t+s,\left(n-1\right)\Delta t+s\right)ds\approx \Delta t\lambda_{i,j}\left(\left(m-0.5\right)\Delta t,\left(n-0.5\right)\Delta t\right)$ . The exponential function could similarly be approximated using the appropriate Taylor polynomial for improved performance.

A first-order scheme for the boundary conditions can be computed as

(25) $$\begin{array}{c} \begin{array}{c} F_{i}^{m,0}=f_{i}\left(m\Delta t,0\right)\\ \;=\sum_{j}\left(\int_{0}^{\infty }\lambda_{j,i}\left(m\Delta t,\tau \right)f_{j}\left(m\Delta t,\tau \right)d\tau \right)\\ \;\approx \sum_{j}\left(\sum_{k>0}\Delta t\lambda_{j,i}\left(m\Delta t,k\Delta t\right)f_{j}\left(m\Delta t,k\Delta t\right)\right)\\ \;=\sum_{k>0,j}\left(\Delta t\lambda_{j,i}\left(m\Delta t,k\Delta t\right)F_{j}^{m,k}\right) \end{array} \end{array}$$

for m≥0 .

One can subsequently estimate the evolution of the random process $X_{t}$ as

(26) $$\begin{array}{c} \begin{array}{c} P\left(X_{t}=i\right)=g_{i}\left(m\Delta t\right)\\ \;\approx \sum_{k=0}^{\infty }\Delta tf_{i}\left(m\Delta t,k\Delta t\right)\\ \;\approx \sum_{k=0}^{\infty }\Delta {\mathrm{tF}}_{i}^{m,k}. \end{array} \end{array}$$

An improvement can be made to this numerical scheme by adjusting the boundary conditions to exactly preserve the total mass at all timesteps:

(27) $$F_{i}^{m,0}=\sum_{j,k}{\tilde{h}}_{j,i}^{m,k}\left(F_{j}^{m-1,k-1}-F_{j}^{m,k}\right)$$

where

(28) $${\tilde{h}}_{i,j}^{m,k}=\frac{\lambda_{i,j}\left(\left(m-\frac{1}{2}\right)\Delta t,\left(k-\frac{1}{2}\right)\Delta t\right)}{\sum_{l}\lambda_{i,l}\left(\left(m-\frac{1}{2}\right)\Delta t,\left(k-\frac{1}{2}\right)\Delta t\right)}.$$

We can then verify that $\sum_{i,k}F_{i}^{m,k}$ is constant; that is, the total probability mass $\sum_{i}g_{i}\left(t\right)$ is conserved. We have here used the midpoint estimates for $\lambda_{i,j}$ . This scheme can improve accuracy when there is a significant scale difference between hazards for competing events.

In epidemiological contexts, hazards are often modeled as continuous and smooth functions, so this scheme will have high accuracy and be convergent due to the inclusion of attractor death states.

A straightforward way to approach the numerical scheme is to calculate $F_{i}^{m,n}$ across all i and all m<0 , tracking the accumulated boundary conditions $F^{m,0}$ for m>0 , and then computing $F_{i}^{m,n}$ for each m≥0 across all i,n . This has been implemented in Supplementary Material: Example Code.

### Hazard Functions and Common Parametric Distributions

The sojourn time density modeling scheme is determined by the hazard rates $\lambda_{i,j}\left(t,\tau \right)$ . It is common to describe these rates parameterically^25,58^ to define a manageable set of model parameters. For example, constant hazard rates $\lambda_{i,j}\left(t,\tau \right)=c$ correspond to an exponential time-to-event distribution, while $\lambda_{i,j}\left(t,\tau \right)=\mathrm{bk}\tau^{k-1}$ and $\lambda_{i,j}\left(t,\tau \right)=pe^{r\tau }$ correspond to Weibull and Gompertz distributions, respectively.^59^ There are many other potential choices for $\lambda_{i,j}\left(t,\tau \right)$ , which may vary with τ , t , or both.

The choice of hazard function is highly dependent on the nature of the system being modeled and the available data sources.^60^ van Wijk et al.^58^ provided a tutorial and general overview of determining appropriate parametrized hazard functions, including periodic functions that can represent seasonal variations or other cyclical patterns. Hazard rates can be estimated from processed survival data, such as smoothed^61^ Kaplan–Meier or Fine–Grey estimates or time-to-event distributions.^36,62^ Covariates can be directly included in hazard rates; for instance, Cox proportional hazard ratios can be used to define ${\tilde{\lambda }}_{i,j}\left(t,\tau \;|\;x\right)=\lambda_{i,j}\left(t,\tau \right)e^{\beta \cdot x}$ for some vector of covariates $x\in R^{n}$ and a baseline hazard function $\lambda_{i,j}\left(t,\tau \right)$ .^41,63^ Constant multipliers for the hazards can be used as calibration targets, to maintain the shape of the distribution.^64^

Asanjarani et al.^36^ showed how time-to-event/discrete event simulation distributions^38,65^ can be translated into hazard rates. Hazard rate can often be defined piecewise,^64^ for example, from life tables.^66,67^ There are also more advanced methods for calculating and representing hazard rates, including nonparametric smoothing^61^ and rational polynomial fraction parametrization.^68^ Arbitrarily detailed and complex hazard rates can be used in sojourn density models; an appropriate level of detail supported by the data must be chosen to avoid overfitting.

### Sojourn-Preserving Transitions

In an epidemiological model, there are often transitions that are independent of the underlying growth of disease. For instance, in a model of cancer development and screening, entry into an “surveillance” cohort would not affect the progress of the disease. This can be reflected in our model framework by introducing transitions that preserve the sojourn time.

To achieve this, we can introduce a new set of hazard rates $\mu_{i,j}\left(t,\tau \right)$ that are the transition rates for an individual in state i to transition to state j , at time t , given that the individual has already spent time τ in the state without any transitions occurring, while conserving the sojourn time. These rates $\mu_{i,j}$ will depend on the type of transition being modeled but would typically have form similar to the $\lambda_{i,j}$ hazard rates (“Hazard Functions and Common Parametric Distributions” section). Then, equation (6) can be modified as follows:

(29) $$\begin{array}{c} \begin{array}{c} \frac{\partial }{\partial t}f_{i}\left(t,\tau \right)+\frac{\partial }{\partial \tau }f_{i}\left(t,\tau \right)=-\left(\sum_{j}\lambda_{i,j}\left(t,\tau \right)\right)f_{i}\left(t,\tau \right)\\ \;-\left(\sum_{j}\mu_{i,j}\left(t,\tau \right)\right)f_{i}\left(t,\tau \right)+\left(\sum_{k}\mu_{k,i}\left(t,\tau \right)f_{k}\left(t,\tau \right)\right) \end{array} \end{array}$$

with boundary condition (7). With this formulation, theorem 2.1 holds with minor alterations.

This approach may be useful for models in which there are multiple categories of states; for example, a health economic model may have a “health outcome” state space {1,2,3,…} (e.g., no lesions, precancerous lesions, cancer) as well as an “intervention” state space {a,b,c...} (e.g., screened, unscreened). The state space corresponding to the Cartesian product of these (1,a),(1,b),(2,a) and so forth can be used for a model that tracks both, with transitions between intervention states governed by the λ parameters and transitions between the intervention states governend by μ .

This can be further extended by continuing to track the sojourn time across multiple independent types of transitions. For instance, one could classify k independent “types” of transitions $\lambda_{i,j}^{1}$ , $\lambda_{i,j}^{2}$ , … $\lambda_{i,j}^{k}$ corresponding to k independent sojourn times $\tau^{1},\tau^{2},...,\tau^{k}$ which are each tracked by the model. Then (29) can be generalized to keep track of multiple sojourn times as

(30) $$\begin{array}{c} \begin{array}{c} \frac{\partial }{\partial t}f_{i}\left(t,\tau \right)+\sum_{k}\frac{\partial }{\partial \tau^{k}}f_{i,j}\left(t,\tau \right)=-\left(\sum_{k,j}\lambda_{i,j}^{k}\left(t,\tau \right)\right)f_{i}\left(t,\tau \right) \end{array} \end{array}$$

where $\tau =\left(\tau^{1},\tau^{2},\ldots ,\tau^{k}\right)$ , with new boundary conditions for each sojourn time $\tau^{i}$

(31) $$f_{i}\left(t,\tau^{1}\left(1-\delta_{1,n}\right),\tau^{2}\left(1-\delta_{2,n}\right),...,\tau^{k}\left(1-\delta_{k,n}\right)\right)=\sum_{k,j}\left(\int_{0}^{\infty }\lambda_{i,j}^{k}\left(t,\tau \right)f_{j}\left(t,\tau \right)d\tau^{k}\right)$$

for n=1,2,…k where $\delta_{x,y}$ is the Kronecker delta function. Then, each transition type $\lambda^{k}$ “sends” the density to the corresponding boundary condition at $\tau^{k}=0$ . This expansion allows for a more general and class of models.

### Computing Health Economic Outcomes

In addition to the likelihood of being in each state, there are other outcomes relevant for health economic analyses that can be computed from these models. These can usually be assessed via either the expected time spent in each state or the likelihood of transition into a state.

For example, the quality-adjusted life-years (QALYs^69^) can be calculated based on the expected time spent in each state. If a person in state i has a QALY value of $x_{i}$ , the expected QALY value over the period $t_{0}$ to $t_{1}$ is the weighted sum

(32) $$\sum_{i}x_{i}\int_{t_{0}}^{t_{1}}g_{i}\left(t\right)\mathrm{dt}.$$

Outcomes such as costs associated with entering a particular state can be calculated via the boundary condition. For instance, the likelihood of entering state i over the period $t_{0}$ to $t_{1}$ is

(33) $$\int_{t_{0}}^{t_{1}}f_{i}(t,0)\mathrm{dt}.$$

If there is a cost $\alpha_{i}$ associated with entering state i , the total expected cost across this period is

(34) $$\sum_{i}\alpha_{i}\int_{t_{0}}^{t_{1}}f_{i}\left(t,0\right)\mathrm{dt}.$$

If there are ongoing costs associated with the time spent in a particular state, these can be calculated in the same way as QALYs above.

### Inhomogeneous Hazard Rates and Infectious Disease Modeling

By relaxing the assumptions on the form of the hazard functions, the sojourn time density models can be used to simulate a more general class of models than semi-Markov models. For example, we could allow the hazard to depend on the current model state, that is, have the form

(35) $$\lambda_{i,j}\left(t,\tau ,g\left(t\right)\right)$$

where $g\left(t\right)=\{g_{i}\left(t\right)\}$ is a vector of the values (4). One can then simulate models where the effect size is dependent on the current state of the system.

For example, the standard SIR model^70^ has states S (susceptible), I (infectious), R (recovered). The risk of infection is proportional to the currently infected population, leading to the hazard

(36) $$\begin{array}{c} \begin{array}{c} \lambda_{S,I}\left(t,\tau ,g\left(t\right)\right)=\beta P\left(X_{t}=I\right)\\ \;=\beta \int_{0}^{\infty }f_{I}\left(t,\tau \right)d\tau \\ \;=\beta g_{I}\left(t\right). \end{array} \end{array}$$

The recovery rate for infected individuals is constant, that is,

(37) $$\lambda_{I,R}\left(t,\tau ,g\left(t\right)\right)=\gamma .$$

These hazards can be used to define a sojourn density model; we can confirm that

(38) $$\frac{dg_{S}}{\mathrm{dt}}=-\beta g_{S}\left(t\right)g_{I}\left(t\right),\frac{dg_{I}}{\mathrm{dt}}=\beta g_{S}\left(t\right)g_{I}\left(t\right)-\gamma g_{I}\left(t\right),\;\frac{dg_{R}}{\mathrm{dt}}=\gamma g_{I}\left(t\right),$$

that is, the defining equations for the standard SIR model. Using this starting point, we could modify this to introduce more complex risks of infection and recovery. For instance, a distribution for time to disease recovery could be simulated by modifying $\lambda_{I,R}$ to be a function of τ , the time in infectious state, to reflect an inflection that can clear after only a few days minimum. In the sojourn density model framework, new dynamics can be included by modifying the hazard functions rather than by adjusting the design of the model.

This addition of heterogeneous generalizes the sojourn density model framework and could allow for the synthesis of different model types into one framework. For example, a model of both a viral infection and a resulting disease (such as hepatitis and liver cancer) could be directly modeled. The addition of proportional terms to the hazard does not affect theorem 2.1 but does mean the method of characteristics described in the Method of Characteristics and survival analysis section may no longer be applied.

### Incorporating Lead-Time Biases

As the sojourn time is modeled explicitly in this framework, this allows us to incorporate lead-time bias. Lead-time biases occur in many contexts; for instance, screen detection of cancer artificially increases survival times due to the increased observation window.^71,72^ A related bias occurs in modeling that has been informed by survival data measured from the start of a trial rather than onset of disease.

These biases can be addressed by modifying the hazard rate. A highly simplistic approach may be to define the adjusted hazard rate

(39) $${\tilde{h}}_{i,j}\left(t,\tau \right)=\left\{ \begin{array}{cc} 0 & \mathrm{for}\;\tau <\tau_{0}\\ \lambda_{i,j}\left(t,\tau -\tau_{0}\right) & \tau \geq \tau_{0} \end{array}\right.$$

where $h_{i,j}\left(t,\tau \right)$ is the observed cause-specific hazard rate from a trial that may include a lead-time bias and $\tau_{0}$ is a cutoff time. This assumes that there is no risk of transition until some given time $\tau_{0}$ . Alternatively, the hazard could be assumed to increase to the observed rates at time $\tau_{0}$ , linearly or otherwise. In practice, a useful estimate for $\tau_{0}$ may be difficult to determine, requiring a sensitivity analysis over a range; there are many useful methods for estimating the lead time.^73–76^

### Example Model: Liver Disease

To demonstrate our new approach to modeling, we now describe a model of liver disease and development of cancer. A key intervention in the prevention of liver cancer death is early detection via routine surveillance. Recommendations like 6-monthly ultrasound for people with cirrhosis (late-stage liver scarring) can increase the likelihood of detection at early stages when curative treatment is possible.^77^ However, people with cirrhosis are also at high risk of both liver decompensation (irreversably worsened liver damage) and noncancer death. People who develop liver decompensation or die before the onset of liver cancer cannot benefit from early detection. It is therefore critical to understand the competing risks of liver cancer, liver decompensation, and other-cause death when assessing whether routine surveillance is advisable or whether the costs and patient discomfort caused by surveillance outweigh the health benefits. This model shows how the risk of liver cancer, and competing risk of decompensation, can be captured in a way that evolves with the length of time spent with liver cirrhosis.

**Figure 2:**
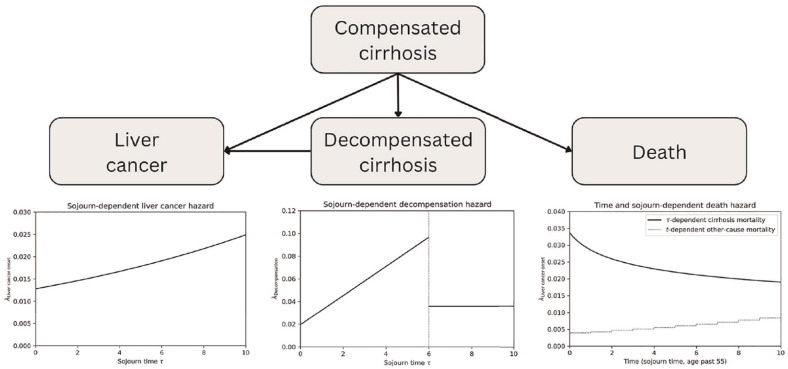
Schematic of the example liver disease model and the sojourn- and time-dependent hazards for competing risks for people with compensated cirrhosis.

The structure of the simplified model is shown in Figure 2 and captures individuals with liver cirrhosis, decompensation events, onset of liver cancer, and death. As the model is designed to illustrate the potential utility of surveillance for liver cancer in the presence of competing risks of decompensation and death, liver cancer and death were used as terminal states. Hazard rates for relevant transitions (decompensation, onset of liver cancer, death) were calibrated by identifying appropriate parametric forms and calibrating to relevant data sources. With this approach, the design of the model states and flow is kept simple, while the complexity is shifted to computing the hazard rates determining the transitions between states. Each cause-specific hazard was fitted in turn, and the overall fit assessed. For this iteration of the model, a cohort aged 55 y was simulated, based on the mean age of cirrhosis diagnosis.

The modeling described in this article is part of a larger model developed to inform the “Clinical Practice Guidelines for Liver Cancer Surveillance for People at High Risk in Australia.”^78^ The development of these guidelines, including the modeling, was led by clinicians in the area of liver disease and liver cancer alongside a multidisciplinary working party, which included health care and clinical representatives, representatives with lived experience, and other community representatives. The full model includes detailed liver cancer stage progression and survival as well as economic details. For further details, see Worthington et al.^79^

For this analysis, published survival curves were used as target data for the modeling. Where patient-level data are available, this could be used as a calibration target instead, using methods in the existing literature on semi-Markov models.^22^

## Results

### Performance of Sojourn Density Models

The described model structure can capture the same level of detail as semi-Markov models and many other patient-level simulations, increasing the level of detail while allowing for deterministic evaluation. The computational complexity of the numerical algorithm described in the “Numerical Scheme” section is $O\left(T^{2}S^{2}\right)$ , where T is the number of timesteps and S is the number of model states. Vectorization across characteristics can improve performance considerably, for example, via the Python NumPy library or other scientific computing libraries. For models in which not every state has a transition to every other state, the complexity is $O\left(T^{2}U\right)$ where $U<S^{2}$ is the number of possible transitions. Compare this with a patient-level simulation, including most implementations of semi-Markov models, in which the computational complexity is driven by the number of patients simulated O(TN) , where N is the number of simulated patients. As noted previously, the precision of such models is approximately $O\left(1/\sqrt{N}\right)$ , and so in some applications, N may need to be very large for precise estimates. This is avoided in the sojourn time density model. For comparison, a standard numerical algorithm for a Markov model would have computational complexity $O\left(TS^{2}\right)$ .

In practice, sojourn density models can run in less than 1 s on a standard computer. This allows for runs with a variety of parameter values to assess sensitivity analyses, particular with parallel computing. In most cases, the optimal timestep Δt can be identified by assessing a particular parameter set, selecting a small enough value for the results of interest to converge. This choice will be highly dependent on the problem being assessed, roughly scaling with the expected transition time. Future work is planned to assess the effect of the timestep.

With an appropriate implementation of the numerical algorithm described in the “Numerical Scheme” section, computing a sojourn density model requires specifying only the model states and the form of the hazard rates between them. Listing 1 shows the syntax of the code for this model; the full code, including the implementation of the numerical scheme, has been included in Supplementary Material: Example Code.

**Listing 1:** Excerpt Showing Code Syntax for the Liver Disease Model^a^

Liver_Disease_Model=Sojourn_Model.Model() state_names=[“Compensated cirrhosis”,“Decompensated cirrhosis”,“Liver ↪ cancer”,“Death”] Liver_Disease_Model.add_state(state_names) *#Liver cancer onset transition* Liver_Cancer_Hazard=Hazard_Functions.Hazard( Hazard_Type = ↪ “gompertz_sojourn”, Parameters={“p”:1.197e-02,“r”:6.654e-02}) Liver_Disease_Model.add_transition(“Compensated cirrhosis”,“Liver ↪ cancer”,Liver_Cancer_Hazard) ///Other transitions omitted for brevity res_df=Liver_Disease_Model.run_model(dt=0.01, max_t=10, ↪ init_conds=[“Compensated cirrhosis”])

a Full code is included in Supplementary Material: Example Code.

### Liver Disease Model Calibration

**Figure 3:**
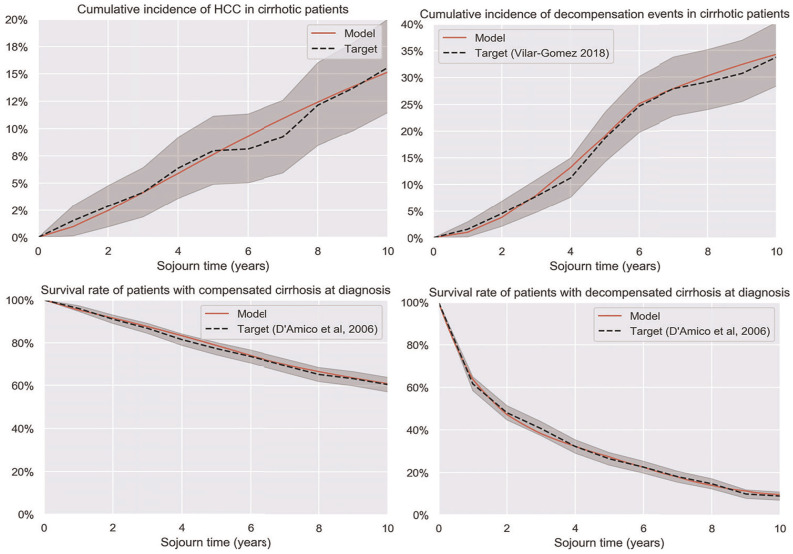
Selected calibration outcomes. Top row: Liver cancer incidence rates (left; target Vilar Gomez et al.^80^) and decompensation rates (right). Bottom row: all-cause death rates in patients with compensated (left; includes any decompensation events) and decompensated (right) cirrhosis.

The model was calibrated to reproduce key targets, with model parameters shown in Table 1. For each target, an appropriate parameterized hazard rate was selected, and optimal parameters were calculated using the BFGS algorithm in the SciPy package. We also calibrated distributions for hazard ratios for each of the hazard rate functions. These were calibrated to reproduce the 95% confidence intervals in each data source across the range of hazard ratio values and act as multipliers for each distribution for the probabilistic sensitivity analysis. The model was well-fitted to the target data, reproducing terminal values for the survival curves and remaining within the 95% confidence intervals for the duration. Key targets are shown in Figure 3. For all-cause death rates, a piecewise constant hazard was used based on age-specific rates published by the Australian Bureau of Statistics.^82^

**Table 1:** Calibrated Parameters for Example Model of Liver Disease

Parameter	Model Value (95% PI)	Target (95% CI)	Note
Cirrhosis decompensation hazard	λ=0.0129t+0.00668fort<6,0.0359fort≥6		Calibrated to Vilar-Gomez et al.^80^
Hazard ratio distribution for PSA	$\mathrm{Lognormal}\;\left(\mu =-0.0213,\sigma^{2}=0.117^{2}\right)$		Calibrated to Vilar-Gomez et al.^80^
Annual decompensation risk (target)	1.03% (0.9%–1.2%)	1.59% (0.17%–3.01%)	Calibration target^80^
Ten-year decompensation risk	34.5% (29.5%–40.2%)	33.7% (28.4%–39.1%)	
Liver cancer onset hazard	$\lambda =0.0102\tau^{1.377}$		Calibrated to Vilar-Gomez et al.^80^
Hazard ratio distribution for PSA	$\mathrm{Lognormal}\;\left(\mu =-0.00171,\sigma^{2}=0.158^{2}\right)$		Calibrated to Vilar-Gomez et al.^80^
Annual liver cancer risk	0.99% (0.8%–1.3%)	1.53% (0.14%–2.93%)	Calibration target^80^
Ten-year liver cancer risk	15.2% (11.8%–19.2%)	15.6% (11.5%–19.7%)	
Death hazard (compensated cirrhosis)	$\lambda =0.0336\tau^{-0.238}$		Calibrated to D’Amico et al.^81^
Hazard ratio distribution for PSA	$\mathrm{Lognormal}\;\left(\mu =-0.0254,\sigma^{2}=0.147^{2}\right)$		Calibrated to D’Amico et al.^81^
Annual death risk (compensated)	4.72% (3.8%–5.8%)	3.97% (2.62%–5.31%)	Calibration target^81^
Ten-year death risk (compensated)	39.4% (35.2%–43.6%)*	39.4% (36.1%–42.8%)	
Death hazard (decompensated cirrhosis)	$\lambda =0.447-0.150\tau +0.0237\tau^{2}$		Calibrated to D’Amico et al.^81^
Hazard ratio distribution for PSA	$\mathrm{Lognormal}\left(\mu =0.0478,\sigma^{2}=0.0895^{2}\right)$		Calibrated to D’Amico et al.^81^
Annual death risk (decompensated)	37.8% (33.1%–42.4%)*	38.3% (35.0%–41.6%)	Calibration target^81^
Ten-year death risk (decompensated)	90.6% (89.0%–91.9%)*	91.1% (89.2%–93.0%)	
Death hazard (other causes)	Age-specific annual rates		Australian life tables^82^

CI, confidence interval; PSA, probabilistic sensitivity analysis.

* 95% PI: 95% prediction interval of model outcomes, based on samples from the specified hazard ratio distributions.

**Figure 4:**
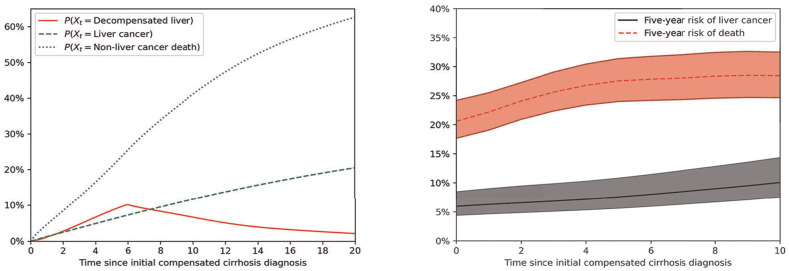
Outcomes from the example model of liver disease. Left: evolving likelihood of liver decompensation, liver cancer, and non–liver cancer death. Right: 5-y risk of liver cancer versus 5-y risk of non–liver cancer death for patients who have not yet developed liver cancer/died, including 95% model range based on probabilistic sensitivity analysis.

A code snippet showing the syntax to define a model using the included code is shown in Listing 1. Examples of the kinds of outputs that can be generated are shown in Figure 4. This includes the likelihood of $X_{t}$ being in a given state by time (through g(t) ). This was used to calculate the 5-y risk of liver cancer or non–liver cancer death in people who have not yet developed liver cancer or died, a key outcome for assessing the benefits of liver surveillance.

A probabilistic sensitivity analysis was run by sampling hazard ratios from the distributions given in Table 1. The sensitivity analysis ran 100,000 parameter sets, which was completed in under 2 h. In the 5-y risk outcomes in Figure 4, this was used to develop a prediction interval; 95% of the simulated parameter sets led to outcomes in the indicated range.

## Discussion

We described a novel approach to epidemiological modeling by calculating the evolution of the probability density of the sojourn time. This allows detailed survival data sources to be used to model competing risks. We demonstrated this sojourn density model approach by presenting a model of liver disease. The model was calibrated and reproduced the targets accurately with low computational burden.

The novel sojourn density model structure allows for flexible analyses that rely on detailed survival data and flexible time scales. This gives advantage over typical epidemiological modeling approaches, improving detail without sacrificing interprability while limiting computational burden. Designing models that reduce computational burden to facilitate probabilistic sensitivity analyses has been identified as a priority for health technology assessments,^32^ often requiring surrogate methods such as meta-modeling/emulators to reduce the reliance on computationally expensive models.^83–86^

By using the hazard rates as parameters, the models can be interpreted directly in the context of source epidemiology studies, and parameter values can be directly inferred from survival data. In the context of liver disease modeling, this means the model can reflect the true health benefits of early-stage diagnosis by accurately capturing disease progression. The ability to numerically evaluate our model at any time scale also allows us to accurately model phenomena such as surveillance intervals, which were not been included in real-world trials with a high degree of precision. Avoiding a fixed time discretization reduces the likelihood of numerical errors if chosen poorly^87^ and improves the ability to capture short time scale events.

There is great potential to refine survival analysis estimates designed to guide policy recommendations,^15^ and it is hoped that this modeling approach allows for the development of straightforward models that incorporate existing data sources in a clear and flexible way, reducing the need for model calibration and minimizing data postprocessing and model design effort. The benefits of this approach are demonstrated in the closeness of fit to the calibration targets (Figure 3) as well as the flexibility to evaluate surveillance at a wide range of intervals.^79^ By providing a numerical implementation, we have also reduced the implementation burden on health economic practitioners, requiring only the specification of the model states and the hazard rates associated with the transitions between them.

The limitations of this model structure include the semi-Markov property; each state transition depends only on the current state, the sojourn time, and the model time. This approach may therefore not be appropriate in contexts where multiple prior events are required to inform future likelihoods.

The liver disease model described here is a part of a larger model of liver disease,^79^ which was used to evaluate surveillance recommendations for the “Clinical Practice Guidelines for Hepatocellular Carcinoma Surveillance for People at High Risk in Australia.”^78^ The flexible sojourn density model allowed us to analyze combinations of surveillance technologies and intervals to analyze more complex surveillance algorithms and optimize these algorithms through iterative design enabled by the fast computation time and high precision.

There are many potential future developments to build on the work presented here. Higher-order approximations for the integrals in (25) and (24) could be used to derive more accurate numerical schemes, which may help when dealing with pathological hazards such as those that are nonsmooth or have discontinuities. It may also be possible to develop spectral methods to approximate solutions without time discretization.^88^ Further work could also use these models to assess the potential impact of lead-time biases, and the loss in precision when these are not considered in modeling. It is hoped that the method described here will be of general use to health economic practitioners.

## Conclusions

To inform health policy and make an impact, epidemiological models must be able to provide reliable, timely, and interpretable results. Sojourn density models can incorporate published survival data and generate competing risk estimates quickly, allowing for simple design and fast calibration. We hope this approach will be of use to modelers in public health.

## Supplemental Material

sj-pdf-1-mdm-10.1177_0272989X251333398 – Supplemental material for Evaluating Semi-Markov Processes and Other Epidemiological Time-to-Event Models by Computing Disease Sojourn Density as Partial Differential Equations Supplemental material, sj-pdf-1-mdm-10.1177_0272989X251333398 for Evaluating Semi-Markov Processes and Other Epidemiological Time-to-Event Models by Computing Disease Sojourn Density as Partial Differential Equations by Joachim Worthington, Eleonora Feletto, Emily He, Stephen Wade, Barbara de Graaff, Anh Le Tuan Nguyen, Jacob George, Karen Canfell and Michael Caruana in Medical Decision Making
