# Supplementary material for: Evaluating Semi-Markov Processes and Other Epidemiological Time-to-Event Models by Computing Disease Sojourn Density as Partial Differential Equations
Source: Med Decis Making. 2025 May 8;45(5):569–86. doi: 10.1177/0272989X251333398 (PMC12166149; doi:10.1177/0272989X251333398)
Supplement: sj-pdf-1-mdm-10.1177_0272989X251333398 – Supplemental material for Evaluating Semi-Markov Processes and Other Epidemiological Time-to-Event Models by Computing Disease Sojourn Density as Partial Differential Equations [file sj-pdf-1-mdm-10.1177_0272989X251333398.pdf]

Appendix A Additional proof of Theorem 2.1

Theorem A.1. *The random process defined by (4) with dynamics defined by (6) and (7) satisfies conditions (2).*

Proof. If the system enters state i at time t^*, i.e. (Y_n, T_n) is realised as (i, t^*) and remains there to at least time $t^* + \tau^*$ (i.e. $T_{n+1} - T_n \geq \tau^*$), then

$$f_j(t, \tau) = \begin{cases} \delta(t - t^* - \tau) & \text{if } i = j \\ 0 & \text{if } i \neq j \end{cases} \tag{40}$$

for all $t \in [t^*, t^* + \tau^*)$ where $\delta(t)$ is the Dirac delta function. Then

$$\begin{aligned} \frac{d}{dt} g_i(t^* + \tau^*) &= \left(\sum_j \left(\int_0^\infty h_{j,i}(t^* + \tau^*, \tau) f_j(t^* + \tau^*, \tau) d\tau \right) \right. \\ &\qquad \left. - \int_0^\infty \left(\sum_j h_{i,j}(t^* + \tau^*, \tau) \right) f_i(t^* + \tau^*, \tau) d\tau \right) \\ &= \left(- \int_0^\infty \left(\sum_j h_{i,j}(t^* + \tau^*, \tau) \right) \delta(\tau^* - \tau) d\tau \right) \\ &= - \sum_j h_{i,j}(t^* + \tau^*, \tau^*) \end{aligned} \tag{41}$$

and for $j \neq i$

$$\frac{d}{dt} g_j(t^* + \tau^*) = h_{i,j}(t^* + \tau^*, t^*). \tag{42}$$

In other words, the likelihood of transition out of state i is proportional to the sum of the hazards $\sum_j h_{i,j}$ and the likelihood of transition $i \to j$ is proportional to $h_{i,j}$. Then

$$\begin{aligned} P(Y_{n+1} = j, T_{n+1} < t + \Delta t \,| Y_n = i, T_n = t - \tau, T_{n+1} \geq t) \\ = g_j(t + \Delta t) \\ \approx g_j(t) + \Delta t \frac{d}{dt} g_j(t) \\ = \Delta t h_{i,j}(t, \tau) \end{aligned} \tag{43}$$

by the above. By taking the limit $\Delta t \to 0$, (2) is satisfied. □
